# Adaptive Design for Phase II/III Platform Trial of Lassa Fever Therapeutics

**DOI:** 10.3201/eid3102.240251

**Published:** 2025-02

**Authors:** Josephine Bourner, Michel Vaillant, Alex Paddy Abdel Salam, Marie Jaspard, Camille Fritzell, Shevin T. Jacob, Tom E. Fletcher, Michael Ramharter, Nnennaya Ajayi, Sylvanus Okogbenin, Cyril Erameh, Donald Grant, Robert Samuels, Oladele Oluwafemi Ayodeji, Armand Sprecher, Bronner P. Gonçalves, Tansy Edwards, Piero Olliaro, the WALC Work Package 2 Working Group

**Affiliations:** University of Oxford, Oxford, UK (J. Bourner, A.P.A. Salam, B.P. Gonçalves, P. Olliaro); Luxembourg Institute of Health, Strassen, Luxembourg (M. Vaillant); Sorbonne Université, Paris, France (M. Jaspard); St Antoine Hospital, Paris (M. Jaspard); The Alliance for International Medical Action, Dakar, Senegal (M. Jaspard); Inserm, Bordeaux, France (C. Fritzell); Liverpool School of Tropical Medicine, Liverpool, UK, S.T. Jacob, T.E. Fletcher); University Medical Center Hamburg-Eppendorf, Hamburg, Germany (M. Ramharter); German Center for Infection Research, Riems, Germany (M. Ramharter); Alex Ekwueme Federal University Teaching Hospital, Abakaliki, Nigeria (N. Ajayi); Irrua Specialist Teaching Hospital, Irrua, Nigeria (S. Okogbenin, C. Erameh); Kenema General Hospital, Kenema, Sierra Leone (D. Grant, R. Samuels); Federal Medical Centre, Owo, Nigeria (O.O. Ayodeji); Médecins sans Frontières, Brussels, Belgium (A. Sprecher); London School of Hygiene and Tropical Medicine, London, UK (T. Edwards)

**Keywords:** Lassa fever, clinical trials, viruses, phase II/III multicenter randomized controlled platform trial

## Abstract

The current recommendation for treating Lassa fever with ribavirin is supported only by weak evidence. Given the persistent effects in areas with endemic transmission and epidemic potential, there is an urgent need to reassess ribavirin and investigate other potential therapeutic candidates; however, a robust clinical trial method adapted to Lassa fever epidemiology has not yet been established. We propose an adaptive phase II/III multicenter randomized controlled platform trial that uses a superiority framework with an equal allocation ratio and accounts for challenges selecting the primary end point and estimating the target sample size by using an interim analysis.

Lassa fever is a viral hemorrhagic fever endemic to parts of West Africa. Each year, high numbers of Lassa fever cases are reported in Nigeria; in 2023, a total of 1,067 confirmed cases were reported and resulted in 189 deaths; the case-fatality ratio (CFR) was 18% (*1*). Annual case numbers are also high in Sierra Leone (*2*) and Liberia (*3*); sporadic cases are reported in Guinea (*4*), Togo (*5*), and Benin (*6*). Modeling, however, suggests that Lassa virus endemicity stretches much further afield, into countries that have, to date, not reported cases of Lassa fever (*7*).

Illness onset is typically characterized by a series of nonspecific signs/symptoms (e.g., fever, headache, vomiting, and abdominal pain) (*8*). Although Lassa is categorized as a hemorrhagic illness, bleeding is reported for only ≈20% of cases (*8*). Other severe complications of Lassa fever include acute kidney injury, encephalopathy, and respiratory distress, although their reporting and detection among Lassa fever patients is inconsistent and highly variable (*9*,*10*). The reported CFR among patients with Lassa fever also varies, reported as 12% by a recent large prospective observational study in Nigeria% (*10*) but up to 60% for other outbreaks in Nigeria and Sierra Leone (*2*,*11*).

Therapeutic options available to treat Lassa fever are limited. Ribavirin, although not licensed for Lassa fever treatment, is commonly featured in clinical management guidelines in combination with supportive care. Currently, 2 active ribavirin treatment regimens are being used for adults (Table 1), and modifications are available for pregnant women and children (*12*), although none have undergone head-to-head evaluation in comparative trials. Use of ribavirin for Lassa fever treatment is based largely on the results of a single clinical trial in Sierra Leone, conducted by McCormick et al. and published in 1986 (*13*). However, serious limitations with the conduct, methods, and analysis of trial data have cast doubt on its effectiveness and raised concerns about the safety of ribavirin therapy for Lassa fever (*14*). Although other observational studies reporting the outcomes of patients receiving ribavirin therapy have been published (*9*,*15*,*16*), limited inference can be made about the generalizability of their results because of potential biases in the retrospective, noncomparative designs.

**Table 1:** Ribavirin regimens for treating adult patients with Lassa fever (*12*)

Regimen, period, days	Dose	Frequency/duration
McCormick		
1 (loading dose)	33 mg/kg (maximum 2.64 g)	Immediately
1–4	16 mg/kg (maximum 1.28 g)	Every 6 h
5–10	8 mg/kg (maximum 0.64 g)	Every 8 h
Irrua		
Day 1 (loading dose)	100 mg/ kg (maximum 7g)	Divided in 2 doses: 2/3 immediately; 1/3 8 h later
2–7	25 mg/kg	Once a day
8–10	12.5 mg/kg	Once a day

Thus, the need to reevaluate ribavirin and explore other potential therapeutic options is urgent. However, clinical research for emerging infectious diseases, such as Lassa fever, is challenging, both methodologically and operationally.

Although Lassa fever is endemic to several West Africa countries, each year patients are hospitalized over a large geographic area (*17*) for which recruitment into a clinical trial would be limited by logistical factors such as access, funding, and human resources. The wide geographic distribution of patients creates further challenges because introduction of heterogeneity may result from variations in Lassa virus strain, illness and death rates, ribavirin availability and use, and supportive care capabilities, which vary across sites and countries.

The limited number of large-scale prospective clinical research studies also makes it difficult to identify a clinically meaningful and measurable primary end point, which is required to generate a sample size that can be feasibly achieved through the available pool of patients and trial sites. Specifically, using death as a primary end point may, in fact, not be viable for Lassa fever treatment trials because the sample size would potentially require recruiting several thousand patients to detect a significant treatment effect (*18*). Identifying an alternative end point that is clinically relevant, however, is complicated by the limited heterogeneous data that have been published to date (*8*).

All those issues make it critical that future clinical trials involving Lassa fever use a standardized method. Furthermore, to accommodate potentially large requisite sample sizes and avoid resource waste and effort duplication, future clinical trials will need to be collaborative: multisite and potentially multicountry. Therefore, a broad range of stakeholders need to agree with regard to key aspects of trial design. Without a co-developed approach, future clinical trials evaluating Lassa fever therapeutics are at risk for being fragmented, underpowered, biased, and difficult to interpret among the countries and sites where they are conducted.

In 2021, the West Africa Lassa Fever Consortium (WALC) was established to move forward therapeutic advances for Lassa fever, from clinical development through availability of and access to effective treatments. The consortium joined >100 stakeholders from public health, academic research, industry, and drug regulation, among other areas, to generate a clinical development plan, including a target product profile for Lassa fever therapeutics, research capacity development plan, clinical trial proposal, and value proposition (*19*). We describe the clinical trial proposal developed by WALC, for which the prepositioned protocol has been published separately (*20*) and the key design considerations were taken into account to ensure that future trials can generate reliable and clinically meaningful results.

## Methods

A consultation group was established to develop a prepositioned protocol for an adaptive phase II/III randomized controlled platform trial to evaluate multiple Lassa fever therapeutics. The consultation group consisted of 56 stakeholders representing clinicians and clinical researchers with experience and expertise in the treatment of Lassa fever, drug developers, ethics committees, nongovernment organizations, public health agencies, regulatory bodies, social scientists, and statisticians (Table 2). Most stakeholders represented organizations based in West Africa (57%); the rest represented pan-African organizations (4%), international organizations (4%), and organizations based outside Africa in Europe and the United States (36%) (Table 2).

**Table 2:** West Africa Lassa fever Consortium stakeholder roles and regions in which their organizations were based

Stakeholder role	Total	No. (%) persons			
		West Africa	Pan-Africa	Outside Africa*	International
Clinical researchers†	15	1 (7)	0	14 (93)	0
Clinician‡	7	7 (100)	0	0	0
Drug developer	3	0	0	3 (100)	0
Ethics committee	2	2 (100)	0	0	0
Nongovernment organization	9	8 (89)	0	0	1 (11)
Public health agency	11	9 (82)	1 (9)	0	1 (9)
Regulatory body	1	0	1 (100)	0	0
Social scientists	5	5 (100)	0	0	0
Statisticians	3	0	0	3 (100)	0
Total	56	32 (57)	2 (4)	20 (36)	2 (4)

*Organizations based in Europe and the United States. †Primary function at their organization was research, but many also held clinical roles. ‡Primary function at their organization was patient management, but all conducted research.

The protocol was developed through group discussion. Remote meetings were scheduled on average every 2 weeks, and 1 face-to-face meeting was held in Abidjan, Côte d’Ivoire, in February 2022 to generate consensus on key design issues. Discussion information came from data available from Lassa fever treatment centers, clinician representatives involved in WALC, and published research data. To supplement the available data, 2 additional research studies were conducted: a systematic review of supportive care guidelines used to provide information for the supportive care requirements described in the protocol (Appendix 1) and a survey of clinicians who work in Lassa fever treatment centers to provide information across sites about the variation in Lassa fever treatment practices and the acceptability of placebo-controlled trials (Appendix 2).

## Results

Among the challenges of designing clinical trials for Lassa fever is the lack of robust clinical data available to use for end point selection and sample size estimation. Furthermore, diverse clinically meaningful outcomes arising in the potential patient pool, combined with sporadic overall case numbers (*8*) and limited available therapeutic options, make enrolling a sufficient number of patients within a reasonable timeframe difficult.

As a result of those issues, a portfolio approach to the design of a phase II/III trial, which enabled the combined evaluation of multiple drug candidates based on their own individual characteristics and requirements through a single platform to optimize research efforts, was selected by using a superiority framework with an equal allocation ratio. To account for uncertainties around the frequencies of the outcomes included in a composite end point, the sample size would initially be calculated for a single end point of death, with a planned interim analysis for sample size reestimation to evaluate or confirm the feasibility of achieving sufficient numbers of patients by using data underpinning the composite end point (Figure). Although the sample size needed to assess a primary end point of death may be large, initiation of the trial would require its acceptance by the research team should the reestimated sample size not decrease after the interim analysis.

**Figure:**
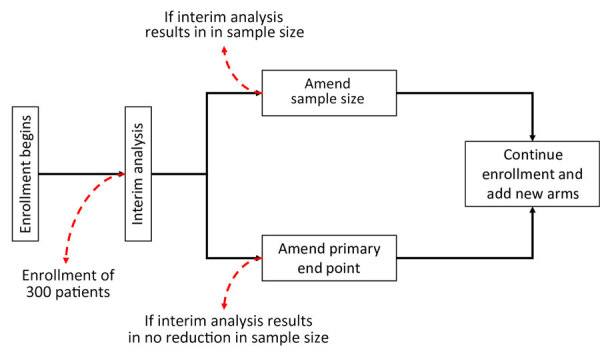
Proposed design of adaptive phase II/III randomized controlled platform trial to evaluate multiple Lassa fever therapeutics.

Our trial design is intended to use the limited number of Lassa fever cases efficiently by enabling data collection for evaluation of multiple therapeutic options to begin in parallel with data collection to validate the end point selection. The trial initially recruits nonpregnant adults and has the potential to expand to pregnant women and children after the safety and efficacy of investigational medicinal products have been established.

### Eligibility Criteria

Eligibility criteria consensus was achieved (Table 3). Two proposals for the enrollment of patients based on diagnostic criteria were considered. The first proposal considered enrollment based on clinical suspicion of Lassa fever, pending reverse transcription PCR (RT-PCR) confirmation. In that scenario, all patients with suspected Lassa fever would be eligible for inclusion, and treatment and would be initiated at the point of enrollment. If the RT-PCR returned a positive result, the patient would continue to receive treatment and remain in the study, but if the RT-PCR returned a negative result, the patient would be withdrawn from treatment and the study. The second proposal considered enrolling patients only after receipt of a positive RT-PCR test result. To minimize risk that persons with suspected cases might be unnecessarily exposed to experimental therapies, the working group ultimately decided, after consultation with drug regulators and clinicians at Lassa fever treatment centers, that patients should be enrolled in trials only after receipt of a positive RT-PCR result confirming Lassa fever diagnosis.

**Table 3:** Criteria for the inclusion or exclusion in future clinical trials for Lassa fever

Criteria
Inclusion criteria: all patients must meet criteria to be included in the trial
Reverse transcription PCR confirmation of Lassa fever
Adult participants (persons who had attained the age of majority according to national regulations in their country of enrollment)
Exclusion criteria: patients who meet any of the criteria will be excluded from the trial
Patients receiving end-of-life care for another illness
Involvement in another clinical trial
Unwilling to provide informed consent
History of allergic reaction or other contraindication to trial drugs
Received drug therapy for Lassa fever (excluding supportive care) before inclusion

### Primary End Point

On the basis of results of a previous exercise, which revealed that evaluating all-cause deaths as a primary end point for Lassa fever would require unfeasible sample sizes (*18*) and a review of data presented in published scientific literature (*9*,*10*), the working group selected “unfavorable outcome” as the primary end point (Table 4). The composite primary end point offered a potential solution to the sample size challenges posed by a single primary outcome measure of all-cause deaths or any other single outcome because the available data revealed no other clinically meaningful outcome that was more frequent than deaths. Basing sample size calculations on the combined event rate of several clinically meaningful outcomes would theoretically decrease the number of patients needed to be enrolled to detect a statistically significant result.

**Table 4:** Composite primary end point for adaptive phase II/III randomized controlled platform trial to evaluate multiple Lassa fever therapeutics

Parameter (any of the following)	Measurement definition	Assessment time point*
Death	1) Yes, 2) No	Days 1–28
New onset of acute kidney injury	KDIGO 3 (https://kdigo.org)	Day 1: hospital discharge
New onset of acute respiratory failure	Arterial oxygen partial pressure/fractional inspired oxygen <315 (*21*) based on 2 consecutive measurements taken >4 h apart meeting the above criteria	
New onset of shock	Mean arterial pressure <65 mm Hg (*22*) based on 2 consecutive measurements taken >4 h apart meeting the above criteria	

*Events will be evaluated on day 1 after treatment initiation.

The composite outcome assesses the new onset of an event any time after initiation of treatment, which patients can meet only 1 time. Any events meeting the criteria (Table 4) that are present at the point of enrollment (and before initiation of treatment) would not be included in the final analysis. If a patient is enrolled with one of the events and another subsequently develops after initiation of treatment, only the second event would be included in the final analysis. Detection of both separate events after the initiation of treatment is equivalent to a single outcome.

### Sample Size and Interim Analysis

A critical problem with the composite end point is the absence of published data for the frequency of the component outcomes to use to calculate the target sample size. Specifically, published data are lacking on patients who experience 2 consecutive recordings of mean arterial pressure <65 mm Hg or 2 consecutive recordings of the ratio of arterial oxygen partial pressure to fractional inspired oxygen (SpO_2_/FiO_2_) >315.

Because of those uncertainties, the sample size calculation will initially conservatively assume that the frequency of composite end point is the same as the all-cause deaths end point. Sample size calculation will therefore be based on a mortality rate of 15% in the control arm (*(**10*,*23**)*), effect size of 33% relative risk reduction (a 10% absolute risk reduction for clinical importance), 90% power, and 10% loss to follow-up, generating a target sample size of 1,010 in each arm, which for a 2-arm trial may take >6 years to achieve.

After enrolling 300 patients, an independent Data Safety and Monitoring Board (DSMB) will conduct an interim analysis evaluating the overall event rate (blind to treatment allocation) to assess the feasibility of achieving a sample size informed by data relating to the outcomes included in the composite end point. The analysis will describe the risk for new onset of the component outcomes of the composite end point at the point of randomization, including their variation by site. That information will be used to reestimate the target sample size, taking into account the precision of the prevalence estimates by using a method selected by the DSMB (*24*). To account for variation in outcomes between health centers, subsequent randomization will be stratified by site. The composite end point will be deemed feasible if the reestimated sample size substantially lowers the target sample size and is attainable within a shorter time. For example, scenarios for projected recruitment times under a new target sample size could be presented to the DSMB along with revised expectations for timing of delivery of further results and treatments for patients. In that instance, the sample size will be adjusted via an amendment, and the trial will continue with the composite end point (Table 4).

If the frequency of the events in the composite end point is not considerably higher than the frequency of deaths in the study population, the trial team will consider the feasibility of continuing the study with either a single end point of death or the composite end point. For example, there is no clear indication that a composite end point will reduce the sample size such that clinically relevant results will be available sooner. To ensure trial integrity (*25*), details about the sample size reestimation and decision process will not be shared with investigators to avoid indirect inferences on the interim data.

### Control Arm

We surveyed clinicians at treatment centers in West Africa about the acceptability of enrolling Lassa fever patients in placebo-controlled trials (Appendix 2). In total, 17 clinicians from 6 health facilities that receive Lassa fever patients in Nigeria, Sierra Leone, and Liberia responded. A total of 59% of respondents stated that they would not enroll their patients in that type of trial, 6% stated that they were not sure, and 12% stated that they would enroll only patients with mild cases.

Separate discussions with representatives of ethics committees and regulators within the consortium revealed similar concerns about randomizing patients to receive supportive care alone. Therefore, ribavirin would need to be used as the control arm treatment. However, there are 2 regimens currently in use for adult patients, for which there is no strong clinical or pharmacokinetic evidence base (*26*,*27*) and that were reported to be in equal use across the treatment centers involved in the survey (Appendix 2). Thus, neither which ribavirin regimen should be used in a control arm nor which regimen would be acceptable to most clinicians involved in a trial is clear. Several options were considered, but resolving the issue in a clinical trial comparing different ribavirin regimens also presents challenges, particularly if randomizing patients to supportive care alone would not be acceptable. In that scenario, establishing the effect of ribavirin on Lassa fever patient outcomes would still be challenging, to the extent that even if 1 regimen demonstrates greater efficacy than another, differentiating between whether that regimen performs better than no active treatment or simply does not harm patients would be difficult. Subsequently interpreting the results of future trials comparing different therapeutic options to the better-performing ribavirin regimen would also be at risk of generating inconclusive results. We present several design options that could be considered (Appendix 3).

### Supportive Care

The systematic review of supportive care guidelines (Appendix 1) returned limited consistent recommendations for the management of acute kidney injury, acute respiratory distress syndrome, and shock; no guidelines met the eligibility criteria describing supportive care for encephalopathy. The working group was, therefore, unable to make an evidence-based recommendation for what should comprise supportive care within the trial.

Moreover, because of variation between supportive care practices and the availability of resources between treatment centers, the trial had to be designed in such a way that heterogeneity in patient outcomes influenced by supportive care practices would not affect evaluation of the treatment effect. Accordingly, the protocol defines only minimum requirements for patient monitoring that can be feasibly implemented across all sites and that will detect the composite primary outcome parameters by using a standardized definition. Ultimately, decisions about supportive care provided in the trial will be made according to local practices at each site.

## Discussion

We describe an adaptive phase II/III randomized controlled platform trial for evaluating multiple therapeutics for Lassa fever that accounts for uncertainties that underlie critical assumptions of the trial design. The aim of WALC was to design a trial that efficiently generates reliable and clinically meaningful results. Forty years have passed since the last treatment trial was conducted, and concerns are growing about the safety of using ribavirin to treat Lassa fever; therefore, there is little time to wait for further observational studies to resolve uncertainties around the frequency of patient outcomes.

The portfolio approach enables concurrent evaluation of multiple therapeutic options and permits efficient and flexible assessment in that it prevents the need for multiple trials to generate evidence across multiple different comparisons, enables data to be collected consistently and in a comparable manner, avoids focus and investment being dedicated to one lead product without overlooking the rest of the pipeline, and reduces time to reach a clinical decision through the existence of an established research infrastructure. Conducting an interim analysis by using data from the first 300 patients randomized to treatment would strengthen the assumptions made in the trial’s primary end point and sample size calculation. In other words, the trial would collect the clinical data needed to accurately reestimate its target sample size for use of the composite end point without having to wait for the results of a separate observational study. The interim analysis arising from that process would then either confirm the viability of using the composite end point over deaths or reject that approach.

Although using the composite end point confers many benefits for the trial design, it is not without its challenges. In particular, working group members were mindful about selecting a composite outcome measure that is both clinically meaningful and reliably measured. The clinical meaningfulness of a single recording of mean arterial pressure of <65 mm Hg that resolves either spontaneously or with minimal fluid therapy is unclear, particularly when no association between hypotension and death has been reported in large observational cohorts (*10*,*23*). Similarly, the clinical relevance of a single SpO_2_/FiO_2_ recording of ≤315 is also unclear because oxygen saturation naturally fluctuates. For that reason, to meet the definitions, the evaluation of SpO_2_/FiO_2_ for acute respiratory failure and mean arterial pressure for shock requires 2 consecutive measurements, taken at least 4 hours apart, that meet the threshold criteria (Table 4).

Encephalopathy was widely considered to be clinically meaningful. Its correlation with death for patients with Lassa fever has been previously demonstrated (*9*), but identifying a suitable reliable measurement instrument was challenging, and diagnostic practices across sites varied widely.

Another challenge of the composite end point is ensuring that patients are correctly classified as being event-free at baseline. A patient with a SpO2/FiO_2_ of 320 at admission in whom SpO_2_/FiO_2_ ≤315 subsequently develops soon after enrollment would be classified as having an unfavorable outcome, but the difference between those 2 values may just be the result of natural fluctuations in vital signs or progression of a pathophysiologic pathway that was already well under way before initiation of an effective antiviral therapy. That issue should, however, be resolved by randomization.

Last, another challenge is determining the appropriate action that should be taken in the event that the interim analysis shows no reduction in sample size when the sample size calculation is based on more robust data on the composite end point. In that scenario, based on predefined parameters established before the start of the trial, the DSMB would need to decide which end point would generate the most clinically meaningful data for evaluation of the therapeutics included in the trial.

There are undoubtedly other aspects that we have not covered in this article but that can be addressed when setting up a trial. For instance, the trial could, as a secondary objective, gather information on other potential outcomes, such as post–acute-phase sequelae through long-term follow-up or outcomes measures on which there was insufficient agreement or evidence (e.g., encephalopathy [(*28*)] and hematologic alterations [(*29*)]). Collectively, those data will help improve the currently limited body of knowledge about clinical manifestations and potential outcome measures of Lassa fever.

With this trial proposal, developed in collaboration with a broad range of stakeholders in the Lassa fever research landscape, WALC aims to catalyze research progress for a disease that has for decades remained dormant. The published protocol is freely available and can be adapted by any research team with funding to initiate a trial (*20*).

Appendix 1 Review of supportive care guidelines for important complications of Lassa fever.

Appendix 2 Ribavirin use across multiple treatment centers in West Africa and the acceptability of placebo-controlled trials.

Appendix 3 Design considerations for a portfolio approach to Lassa fever clinical trials.
