# Supplementary material for: Adaptive Design for Phase II/III Platform Trial of Lassa Fever Therapeutics
Source: Emerg Infect Dis. 2025 Feb;31(2):e240251. doi: 10.3201/eid3102.240251 (PMC11845141; doi:10.3201/eid3102.240251)
Supplement: Appendix 1 — Review of supportive care guidelines for important complications of Lassa fever. [file 24-0251-Techapp-s1.pdf]

Article DOI: https://doi.org/10.3201/eid3102.240251

EID cannot ensure accessibility for supplementary materials supplied by authors. Readers who have difficulty accessing supplementary content should contact the authors for assistance.

Adaptive Design for Phase II/III Platform Trial of Lassa Fever Therapeutics

Appendix 1

A review of supportive care guidelines for important complications of Lassa fever

There are currently no evidence-based supportive care guidelines (SCGs) that have been developed specifically for Lassa fever and there is a limited number of evidence-based SCGs available for Viral Hemorrhagic Fevers (VHFs).

Definition of evidence-based supportive care guidelines: We define evidence-based supportive care guidelines as guidelines that have been developed using a systematic and transparent review process to assess the available evidence and make clinical practice recommendations. A rationale for each recommendation included in the guideline should be provided with references to each source of evidence.

To limit heterogeneity in patient care, it will be imperative for a multi-center clinical trial for Lassa fever to standardise supportive care practices and implement harmonised SCGs.

To identify evidence-based SCGs that may be appropriate for use in a Lassa fever clinical trial, we conducted a review of existing supportive care guidelines for four key complications of Lassa fever – acute respiratory distress syndrome (ARDS), acute kidney injury (AKI), shock and encephalopathy.

Methods

The aim of this review is to identify existing SCGs that could support a multi-center clinical trial in West Africa and summarize the recommendations that could be used to standardise supportive care in a trial protocol.

We conducted a search of EMBASE, MEDLINE, Global Health, CINAHL, Scopus, and Web of Science Core Collection for SCGs, which were screened against the criteria in Appendix 1 Table 1.

No language restrictions were applied.

Screening was performed by one reviewer. The final list of included studies was reviewed by a second reviewer with clinical expertise in the provision of supportive care to patients with high consequence infectious diseases. Data were extracted by one reviewer on a standardised data collection form in Excel. The results are presented as a descriptive summary of the guidelines for each complication of interest.

Results

A total of 23 SCGS were eligible for inclusion in this review (Appendix 1 Table 2) which included 14 SCGs describing the management of shock, seven SCGs describing the management of ARDS, and two SCGs describing the management of AKI. No SCGs meeting the above eligibility criteria were identified for the management of encephalopathy.

Of the 23 included SCGs, 11 included recommendations for children, eight included recommendations for pregnant women, and five included (or were developed exclusively for) settings with limited resources.

Acute respiratory distress syndrome (ARDS)

Six of the seven included SCGs were developed for the management of ARDS in adults and one was developed for children. None of the included guidelines were developed specifically for or made provision for pregnant women or patients with VHFs.

Detection

Of the seven included SCGs for ARDS four provided a definition of ARDS (*1–4*) and used the Berlin definition (*5*) (Appendix 1 Table 2**).** This definition however requires arterial blood gas measurement, which may not be available in some Lassa fever treatment centers. In these settings, the detection of ARDS could be facilitated by the "Kigali modification" to the Berlin definition (*6*).

Management

Four guidelines made recommendations related to mechanical ventilation (*2–4*) and high flow nasal oxygen (HFNO) (*7*). Equipment to support these recommendations may however be limited in Lassa fever treatment centers.

One guideline made specific recommendations for fluid therapy (*1*), stating fluid restriction is preferable over a liberal fluid strategy in adults with ARDS.

Four guidelines made recommendations for prone positioning in ARDS (*2–4*,*8*), of which two guidelines made recommendations for the duration of prone positioning for ventilated patients: at least 12 hours per day for patients with severe ARDS (*3*); 16 hours per day in the first week of moderate to severe ARDS (*2*). One guideline recommended prone positioning for patients with ARDS only when the PaO2/FIO2 ratio <150 mm Hg (*4*). One guideline recommended that prone positioning should not be part of routine therapy in pediatric ARDS (PARDS), but stated that it should be considered an option in cases of severe PARDS (*8*).

Three guidelines made recommendations for recruitment procedures (*2–4*). One guideline made a favorable recommendation for the use of recruitment procedures in adults (*3*), while the two others recommended its use only as a rescue measure in catastrophic hypoxaemia (*4*), in cases of clear derecruitment, or refractory hypoxemia (PaO2/FiO2 <100 mm Hg) despite optimization of therapy and in the absence of contraindication (*2*).

Drug therapies

Three guidelines recommended specific drug therapies to be used in ARDS (*1*,*4*,*8*).

Inhaled nitric oxide (iNO) was not recommended for routine use in ARDS in any of the three guidelines. Two guidelines recommended iNO in cases of “deep hypoxemia” (which was qualified in one guideline by: despite the implementation of a protective ventilation strategy and prone positioning and before envisaging use of venovenous ECMO) (*1*,*4*). One guideline recommended iNO in children with documented pulmonary hypertension or severe right ventricular dysfunction as a rescue from or bridge to extracorporeal life support (*8*).

One guideline recommended neuromuscular blocking agents (NMBAs), in which NMBAs were suggested for early use in adults during the early stages of severe ARDS (*1*).

Two guidelines made a recommendation against corticosteroids (*1*,*8*). In routine care, recommendations were also made against Beta-2 antagonists, prostanoids, and statins in adults (*1*), and helium-oxygen mixture, inhaled or IV prostaglandins therapy, plasminogen activators, fibrinolytics, or other anticoagulants, inhaled β-adrenergic receptor agonists or ipratropium, IV N-acetylcysteine for antioxidant effects or intratracheal N-acetylcysteine for mobilizing secretions, dornase α outside of the cystic fibrosis population, and a cough assist device in children (*8*).

One guideline stated that outside the scenarios in which iNO and NBAs were recommended, no other drugs should be used as part of routine care for ARDS (*1*).

Acute kidney injury (AKI)

Both guidelines were developed for use in adults (*9*,*10*) and one guideline included pediatric considerations (*10*). Neither of the included guidelines were developed specifically for or made provision for pregnant women or VHF patients.

Detection

Both SCGs recommended detection of AKI using serum creatinine (*9*,*10*) or urine volume (*10*). In a multi-center trial where laboratory capacity varies between sites, the KDIGO guideline recommendation for detection of AKI may be more appropriate allowing for either serum creatinine measurement or urine volume measurement.

Management

Both guidelines recommended crystalloid fluid to manage AKI (*9*,*10*). Crystalloid fluids were considered to be as safe and effective as using hypooncotic colloids (*9*) and preferred over colloids in the absence of hemorrhagic shock (*10*).

One guideline recommended a target Mean Arterial Pressure (MAP) of ≥65 mm Hg (*9*).

While several recommendations were made for the use of RRT, few Lassa fever treatment centers would have access to sufficient numbers of dialysis machines, nephrologists or nephrology nurses to safely and consistently incorporate a standardised recommendation in to a trial protocol.

Glycaemic control and nutritional support was discussed in one guideline (*10*).

Recommendations for target urine output were not noted in either guideline.

The use of diuretics was not recommended, except to test renal responsiveness after adequate fluid loading (followed by discontinuation if there is insufficient response) (*9*) and in the management of fluid overload (*10*).

Drug therapies

The use of vasopressors was recommended by both guidelines (*9*,*10*). Although neither guideline stated a first choice of vasopressor nor did they specify an escalation strategy.

Insulin therapy targeting plasma glucose 110–149 mg/dl (6.1–8.3 mmol/l) was suggested for critically ill patients (*10*). Nutrition was suggested via the enteral route in patients with AKI. Nutritional support suggestions included achieving a total energy intake of 20–30 kcal/kg/d in patients with any stage of AKI, avoiding restriction of protein intake with the aim of preventing or delaying initiation of RRT, administering 0.8–1.0 g/kg/d of protein in non-catabolic AKI patients without need for dialysis, 1.0–1.5 g/kg/d in patients with AKI on RRT, and up to a maximum of 1.7 g/kg/d in patients on CRRT and in hypercatabolic patients (*10*).

Shock

Of the 14 included SCGs for shock, nine were developed for use in adults (*11–19*), of which four included provisions for children (*13*,*14*,*16*,*18*) and a further three were developed specifically for children (*20–22*).

One guideline was developed for the management of shock in pregnancy (*23*).

Five guidelines were developed for use in resource limited settings.

Management

Three guidelines specified a target systolic blood pressure of ≥90mm Hg for adult patients (*12*,*18,23*), of which one guideline was written for use in pregnant women (*23*). One guideline recommended individualising the target blood pressure during shock resuscitation and tolerating a lower level of blood pressure in patients with uncontrolled bleeding without severe head injury (*11*). None stated targets for children.

Six guidelines made a recommendation for target Mean Arterial Pressure (MAP) (*11–13*,*16*,*19,23*). In adults, MAP ≥65 mm Hg was recommended as a target in five guidelines (*11–*

13,*16*,*19*) and, for pregnant women, MAP of ≥70 mm Hg was identified as 'normal' according to obstetrically modified SOFA score (omSOFA) (*23*). One guideline suggested using a higher MAP in septic patients with a history of hypertension and those showing clinical improvement with higher blood pressure (*11*).

Two guidelines made a recommendation for target lactate: <2 mmol/L in non-pregnant adults (*16*) and 0.6 – 1.8 in pregnant women according to the omSOFA (*23*).

Eight guidelines stated a recommendation for blood pressure monitoring and (*11–13*,*16*,*18–20,22*) five guidelines made a recommendation for cardiac output monitoring (*11*,*16*,*19,20,22*). However, one guideline did not recommend that routine monitoring of cardiac output was not needed in patient with shock who respond to initial therapy (*11*). In resource-limited settings, the passive leg raise test was recommended as an acceptable method for measuring cardiac output (*16*).

Eight guidelines recommended crystalloid fluids for fluid resuscitation (*12*,*13*,*16*,*19,21–24*) and two guidelines recommended that either crystalloid or colloids could be used (*18,20*).

In resource-limited settings (with either no or limited access to vasopressors, mechanical ventilation or intensive care), a conservative fluid resuscitation strategy or avoidance of bolus fluid resuscitation was recommended (*16*,*21,22*), along with stopping fluid administration if patients develop signs of respiratory distress or lung crepitations (*16*).

Drug therapies

Five guidelines described the criteria for initiating inotropic and/or vasopressor therapy (*11*,*13*,*16*,*18,20*) and seven guidelines made recommendations for first-line therapy (*12*,*13*,*16*,*18–20,22*).

Eight guidelines made a recommendation for adjunct steroids in the treatment of shock in adults (*13–15*,*18–20,22,23*). In the majority of cases the use of steroids was qualified by specific conditions that should be met before administration.

Other therapies were also recommended in all guidelines depending on the affected population, suspected cause of shock and complications.

Discussion

Identifying a single SCG or detecting widespread agreement on specific aspects of the management across several SCGs for use in a trial protocol is challenging due to the varying foci of the included SCGs and anticipated varying resource availability and local practices across multiple health centers in West Africa.

Resource availability and local practice is a critical consideration in a multi-center clinical trial, in which supportive care would need to be protocolised both safely – ensuring the sufficient availability of qualified staff and that equipment is maintained appropriately – and consistently – ensuring equivalence in equipment and staff training – to limit heterogeneity in patient outcomes between treatment centers. In settings with limited existing staff and equipment resource, standardising the management of ARDS and AKI, for example, is challenging as both complications require substantial equipment investments, infrastructure support and specialist staff training; the cost of which will be prohibitively expensive for a clinical trial.

Altering current clinical practice to standardise processes across multiple trial sites may also be unacceptable (for example, the criteria for initiating RRT or first-line drug therapies) and therefore infeasible.

As a result of these important challenges the WALC agreed to focus on the detection and monitoring of the ARDS, AKI, shock and encephalopathy, which would not interfere with local clinical practice and decision-making, and would not require substantial financial investments. As it was not possible to identify a single existing SCG or recommendation for which there was widespread agreement, further consultation took place within the consortium to standardise the detection and monitoring of the abovementioned complications.

References

1. Claesson J, Freundlich M, Gunnarsson I, Laake JH, Møller MH, Vandvik PO, et al. Scandinavian clinical practice guideline on fluid and drug therapy in adults with acute respiratory distress syndrome. Acta Anaesthesiol Scand. 2016;60:697–709. PubMed https://doi.org/10.1111/aas.12713

2. Claesson J, Freundlich M, Gunnarsson I, Laake JH, Vandvik PO, Varpula T, et al.; Scandinavian Society of Anaesthesiology and Intensive Care Medicine. Scandinavian clinical practice guideline

on mechanical ventilation in adults with the acute respiratory distress syndrome. Acta Anaesthesiol Scand. 2015;59:286–97. PubMed https://doi.org/10.1111/aas.12449

3. Fan E, Del Sorbo L, Goligher EC, Hodgson CL, Munshi L, Walkey AJ, et al.; American Thoracic Society, European Society of Intensive Care Medicine, and Society of Critical Care Medicine. An Official American Thoracic Society/European Society of Intensive Care Medicine/Society of Critical Care Medicine Clinical Practice Guideline: Mechanical Ventilation in Adult Patients with Acute Respiratory Distress Syndrome. Am J Respir Crit Care Med. 2017;195:1253–63. PubMed https://doi.org/10.1164/rccm.201703-0548ST

4. Papazian L, Aubron C, Brochard L, Chiche J-D, Combes A, Dreyfuss D, et al. Formal guidelines: management of acute respiratory distress syndrome. Ann Intensive Care. 2019;9:69. PubMed https://doi.org/10.1186/s13613-019-0540-9

5. Ranieri VM, Rubenfeld GD, Thompson BT, Ferguson ND, Caldwell E, Fan E, et al.; ARDS Definition Task Force. Acute respiratory distress syndrome: the Berlin Definition. JAMA. 2012;307:2526–33. PubMed

6. Riviello ED, Kiviri W, Twagirumugabe T, Mueller A, Banner-Goodspeed VM, Officer L, et al. Hospital incidence and outcomes of the acute respiratory distress syndrome using the Kigali modification of the Berlin definition. Am J Respir Crit Care Med. 2016;193:52–9. PubMed https://doi.org/10.1164/rccm.201503-0584OC

7. Rochwerg B, Einav S, Chaudhuri D, Mancebo J, Mauri T, Helviz Y, et al. The role for high flow nasal cannula as a respiratory support strategy in adults: a clinical practice guideline. Intensive Care Med. 2020;46:2226–37. PubMed https://doi.org/10.1007/s00134-020-06312-y

8. Tamburro RF, Kneyber MC; Pediatric Acute Lung Injury Consensus Conference Group. Pulmonary specific ancillary treatment for pediatric acute respiratory distress syndrome: proceedings from the Pediatric Acute Lung Injury Consensus Conference. Pediatr Crit Care Med. 2015;16(Suppl 1):S61–72. PubMed https://doi.org/10.1097/PCC.0000000000000434

9. Brochard L, Abroug F, Brenner M, Broccard AF, Danner RL, Ferrer M, et al.; ATS/ERS/ESICM/SCCM/SRLF Ad Hoc Committee on Acute Renal Failure. An official ATS/ERS/ESICM/SCCM/SRLF statement: Prevention and Management of Acute Renal Failure in the ICU Patient: an international consensus conference in intensive care medicine. Am J Respir Crit Care Med. 2010;181:1128–55. PubMed https://doi.org/10.1164/rccm.200711-1664ST

10. Khwaja A. KDIGO clinical practice guidelines for acute kidney injury. Nephron Clin Pract. 2012;120:c179–84. PubMed https://doi.org/10.1159/000339789

11. Cecconi M, De Backer D, Antonelli M, Beale R, Bakker J, Hofer C, et al. Consensus on circulatory shock and hemodynamic monitoring. Task force of the European Society of Intensive Care Medicine. Intensive Care Med. 2014;40:1795–815. PubMed https://doi.org/10.1007/s00134-014-3525-z

12. Chioncel O, Parissis J, Mebazaa A, Thiele H, Desch S, Bauersachs J, et al. Epidemiology, pathophysiology and contemporary management of cardiogenic shock - a position statement from the Heart Failure Association of the European Society of Cardiology. Eur J Heart Fail. 2020;22:1315–41. PubMed https://doi.org/10.1002/ejhf.1922

13. Dellinger RP, Levy MM, Rhodes A, Annane D, Gerlach H, Opal SM, et al.; Surviving Sepsis Campaign Guidelines Committee including the Pediatric Subgroup. Surviving sepsis campaign: international guidelines for management of severe sepsis and septic shock: 2012. Crit Care Med. 2013;41:580–637. PubMed https://doi.org/10.1097/CCM.0b013e31827e83af

14. Lamontagne F, Rochwerg B, Lytvyn L, Guyatt GH, Møller MH, Annane D, et al. Corticosteroid therapy for sepsis: a clinical practice guideline. BMJ. 2018;362:k3284. PubMed https://doi.org/10.1136/bmj.k3284

15. Mer M, Schultz MJ, Adhikari NK; European Society of Intensive Care Medicine (ESICM) Global Intensive Care Working Group and the Mahidol–Oxford Research Unit (MORU), Bangkok, Thailand. Core elements of general supportive care for patients with sepsis and septic shock in resource-limited settings. Intensive Care Med. 2017;43:1690–4. PubMed https://doi.org/10.1007/s00134-017-4831-z

16. Misango D, Pattnaik R, Baker T, Dünser MW, Dondorp AM, Schultz MJ; Global Intensive Care Working Group; of the European Society of Intensive Care Medicine (ESICM) and the Mahidol Oxford Tropical Medicine Research Unit (MORU) in Bangkok, Thailand. Haemodynamic assessment and support in sepsis and septic shock in resource-limited settings. Trans R Soc Trop Med Hyg. 2017;111:483–9. PubMed https://doi.org/10.1093/trstmh/try007

17. Serpa Neto A, Schultz MJ, Festic E. Ventilatory support of patients with sepsis or septic shock in resource-limited settings. Intensive Care Med. 2016;42:100–3. PubMed https://doi.org/10.1007/s00134-015-4070-0

18. Dünser MW, Festic E, Dondorp A, Kissoon N, Ganbat T, Kwizera A, et al.; Global Intensive Care Working Group of European Society of Intensive Care Medicine. Recommendations for sepsis management in resource-limited settings. Intensive Care Med. 2012;38:557–74. PubMed https://doi.org/10.1007/s00134-012-2468-5

19. Rhodes A, Evans LE, Alhazzani W, Levy MM, Antonelli M, Ferrer R, et al. Surviving Sepsis Campaign: International Guidelines for Management of Sepsis and Septic Shock: 2016. Intensive Care Med. 2017;43:304–77. PubMed https://doi.org/10.1007/s00134-017-4683-6

20. Davis AL, Carcillo JA, Aneja RK, Deymann AJ, Lin JC, Nguyen TC, et al. American College of Critical Care Medicine Clinical Practice Parameters for Hemodynamic Support of Pediatric and Neonatal Septic Shock. Crit Care Med. 2017;45:1061–93. PubMed https://doi.org/10.1097/CCM.0000000000002425

21. Musa N, Murthy S, Kissoon N. Pediatric sepsis and septic shock management in resource-limited settings. Intensive Care Med. 2016;42:2037–9. PubMed https://doi.org/10.1007/s00134-016-4382-8

22. Weiss SL, Peters MJ, Alhazzani W, Agus MSD, Flori HR, Inwald DP, et al. Surviving Sepsis Campaign International Guidelines for the Management of Septic Shock and Sepsis-Associated Organ Dysfunction in Children. Pediatr Crit Care Med. 2020;21:186–95. https://doi.org/10.1097/PCC.0000000000002197

23. Bowyer L, Robinson HL, Barrett H, Crozier TM, Giles M, Idel I, et al. SOMANZ guidelines for the investigation and management sepsis in pregnancy. Aust N Z J Obstet Gynaecol. 2017;57:540–51. PubMed https://doi.org/10.1111/ajo.12646

24. Perner A, Junttila E, Haney M, Hreinsson K, Kvåle R, Vandvik PO, et al.; Scandinavian Society of Anaesthesiology and Intensive Care Medicine. Scandinavian clinical practice guideline on choice of fluid in resuscitation of critically ill patients with acute circulatory failure. Acta Anaesthesiol Scand. 2015;59:274–85. PubMed https://doi.org/10.1111/aas.12429

Appendix 1 Table 1. Eligibility criteria

Criteria
Inclusion criteria
Evidence-based SCGs making recommendations for the management of acute kidney injury (AKI), acute respiratory distress syndrome (ARDS), shock and encephalopathy
Published since 2010
Developed through international collaboration (either collaboration by institutions based in two separate countries or more; or by an international organization i.e., an organization based in one country with representatives in multiple countries)
Exclusion criteria
Editorials, commentaries and review of SCGs

Appendix 1 Table 2. Summary of included guidelines

Organisation or society responsible for developing the SCG	Title of SCG	Year of publication	Supportive care target
European Society of Intensive Care Medicine	Consensus on circulatory shock and hemodynamic monitoring. Task force of the European Society of Intensive Care Medicine	2014	Shock
Heart Failure Association of the European Society of Cardiology	Epidemiology, pathophysiology and contemporary management of cardiogenic shock – a position statement from the Heart Failure Association of the European Society of Cardiology	2020	Shock
American College of Critical Care Medicine	American College of Critical Care Medicine Clinical Practice Parameters for Hemodynamic Support of Pediatric and Neonatal Septic Shock	2017	Shock
Surviving Sepsis Campaign Guidelines Committee	Surviving Sepsis Campaign: International Guidelines for Management of Severe Sepsis and Septic Shock: 2012	2013	Shock
MAGIC group and the BMJ	Corticosteroid therapy for sepsis: a clinical practice guideline	2018	Shock
European Society of Intensive Care Medicine (ESICM) Global Intensive Care Working Group and the Mahidol–Oxford Research Unit (MORU), Bangkok, Thailand	Core elements of general supportive care for patients with sepsis and septic shock in resource-limited settings	2017	Shock
European Society of Intensive Care Medicine and the Mahidol Oxford Tropical Medicine Research Unit (MORU)	Hemodynamic assessment and support in sepsis and septic shock in resource-limited settings	2017	Shock
European Society of Intensive Care Medicine (ESICM) Global Intensive Care Working Group and the Mahidol–Oxford Research Unit (MORU)	Pediatric sepsis and septic shock management in resource-limited settings	2016	Shock
'European Society of Intensive Care Medicine (ESICM) Global Intensive Care' working group and the 'Mahidol-Oxford Research Unit' (MORU)	Ventilatory support of patients with sepsis or septic shock in resource-limited settings	2016	Shock
Scandinavian Society of Anaesthesiology and Intensive Care Medicine	Scandinavian clinical practice guideline on choice of fluid in resuscitation of critically ill patients with acute circulatory failure	2015	Shock
Surviving sepsis campaign guideline committee	Surviving Sepsis Campaign: International Guidelines for Management of Sepsis and Septic Shock: 2016	2017	Shock
Society of Critical Care Medicine, the European Society of Intensive Care Medicine, and the World Federation of Pediatric Intensive and Critical Care Societies	Surviving Sepsis Campaign International Guidelines for the Management of Septic Shock and Sepsis-Associated Organ Dysfunction in Children	2020	Shock
Society of Obstetric Medicine Australia and New Zealand	SOMANZ guidelines for the investigation and management sepsis in pregnancy	2017	Shock
Global Intensive Care Working Group of European Society of Intensive Care Medicine	Recommendations for sepsis management in resource-limited settings	2012	Shock
Scandinavian Society of Anaesthesiology and Intensive Care Medicine (SSAI)	Scandinavian clinical practice guideline on mechanical ventilation in adults with the acute respiratory distress syndrome	2015	ARDS
Société de Réanimation de Langue Française (SRLF)	Formal guidelines: management of acute respiratory distress syndrome	2019	ARDS
Society of Critical Care Medicine and the World Federation of Pediatric Intensive and Critical Care Societies	Pulmonary Specific Ancillary Treatment for Pediatric Acute Respiratory Distress Syndrome: Proceedings From the Pediatric Acute Lung Injury Consensus Conference	2015	ARDS
American Thoracic Society/European Society of Intensive Care Medicine/Society of Critical Care Medicine	An Official American Thoracic Society/European Society of Intensive Care Medicine/Society of Critical Care Medicine Clinical Practice Guideline: Mechanical Ventilation in Adult Patients with Acute Respiratory Distress Syndrome	2017	ARDS
Scandinavian Society of Anaesthesiology and Intensive Care Medicine (SSAI)	Scandinavian clinical practice guideline on fluid and drug therapy in adults with acute respiratory distress syndrome	2016	ARDS
European Respiratory Society/American Thoracic Society	Official ERS/ATS clinical practice guidelines: noninvasive ventilation for acute respiratory failure	2017	ARDS

Organisation or society responsible for developing the SCG	Title of SCG	Year of publication	Supportive care target
European Society of Intensive Care Medicine	The role for high flow nasal cannula as a respiratory support strategy in adults: a clinical practice guideline	2020	ARDS
American Thoracic Society Documents	An Official ATS/ERS/ESICM/SCCM/SRLF Statement: Prevention and Management of Acute Renal Failure in the ICU Patient	2010	AKI
Kidney Disease: Improving Global Outcomes (KDIGO)	KDIGO Clinical Practice Guideline for Acute Kidney Injury	2012	AKI
