# Supplementary material for: Adaptive Design for Phase II/III Platform Trial of Lassa Fever Therapeutics
Source: Emerg Infect Dis. 2025 Feb;31(2):e240251. doi: 10.3201/eid3102.240251 (PMC11845141; doi:10.3201/eid3102.240251)
Supplement: Appendix 2 — Ribavirin use across multiple treatment centers in West Africa and the acceptability of placebo-controlled trials. [file 24-0251-Techapp-s2.pdf]

Article DOI: https://doi.org/10.3201/eid3102.240251

EID cannot ensure accessibility for supplementary materials supplied by authors. Readers who have difficulty accessing supplementary content should contact the authors for assistance.

Adaptive Design for Phase II/III Platform Trial of Lassa Fever Therapeutics

Appendix 2

Ribavirin use across multiple treatment centers in West Africa and the acceptability of placebo-controlled trials

As the only treatment currently recommended for Lassa fever (LF), ribavirin is a likely candidate to feature as the control arm treatment in a future LF clinical trial. However, multiple ribavirin treatment regimens exist. It is therefore necessary to identify one regimen that could be implemented across all sites participating in a trial.

Methods

To understand the use of ribavirin and perspectives about placebo-controlled trials among clinicians working at LF treatment in West Africa, we conducted a short survey.

The eligibility criteria for participation in the survey was as follows:

- [In countries regularly reporting cases of LF and with dedicated LF treatment centers]: Doctors working in LF treatment centers with first-hand experience administering ribavirin for LF
- [Countries with low reporting of LF or without dedicated LF treatment centers:] Doctors who, if a case of LF presented at their hospital, would be responsible for patient management AND who are familiar with ribavirin regimens for LF

Clinicians were identified to respond to the questionnaire by identifying focal points in Lassa fever-endemic countries to disseminate to their local contacts.

A summary of the responses to the questionnaire has been provided below.

Results

In total, 17 responses were received, of which 14 were from Nigeria, 2 were from Sierra Leone and 1 was from Liberia. The respondents represented clinical staff – 15 doctors and two nurses – from six health centers, all of whom had a minimum of at least 2 years' experience treating patients with LF and more than half had treated over 100 LF patients in their career.

All respondents indicated that ribavirin was available at their health facility to treat LF, of whom 14 respondents (82%) stated both oral and intravenous formulations were available at their facility. The remaining 3 respondents (18%) were not aware which formulations were available.

Of the 15 respondents (88%) who were aware of how ribavirin is sourced at their health facility, 10 (69%) indicated that ribavirin is provided by local or national government agencies. The remaining 5 respondents indicated that ribavirin is provided by NGOs alone or a mix of government agencies and NGO support.

When asked why they use ribavirin to treat Lassa fever, 41% stated that they use ribavirin in accordance with local or national LF case management guidelines, 34% stated they had personal experience of ribavirin being effective therapy for LF, and 25% of respondents indicated that they believe there is good quality evidence that ribavirin improves patient outcomes.

Of the two ribavirin regimens that are commonly used to treat LF in non-pregnant adults, 50% indicated they used the McCormick/WHO regimen and 50% indicated they used the Irrua regimen. The McCormick/WHO regimen was more commonly used for pediatric cases of LF and for pregnant women with LF (85% of respondents used this regimen to treat children and 60% respondents used this regimen to treat pregnant women).

When respondents were asked if they would enrol patients to a clinical trial that used supportive care alone in the control arm, 59% stated they would not be willing to enrol patients at all, 23% stated they would only enrol mild cases, 12% said they would be willing to enrol patients, and 6% stated they were not sure.

Of those who stated they would not be willing to enrol patients to this type of trial, 23% of respondents stated each of the following circumstances under which they would change their mind:

- If new or existing data raises the possibility that ribavirin may be harmful in LF patients
- If a trial is approved by the national ethics committee
- If new or existing data raises the possibility that ribavirin may not be effective in LF patients
- If there is robust supportive treatment available to all patients

One respondent stated that it would never be acceptable to use supportive care alone.

Discussion

The most complex decision for a future Phase III trial for LF therapeutics will be the selection of the control arm intervention. It is clear that using supportive care alone may not be widely accepted by clinicians who would enrol patients to trials, risking low recruitment to a trial which may anyway face challenges achieving its target sample size.

While ribavirin is commonly used and widely available at the treatment centers surveyed within the scope of this exercise, there is variation in the regimens used to treat cases of LF – which appears to be due partly to preference and to direction by local case management guidelines. Any Phase III trial that uses ribavirin in its control arm would need to select one of the two available regimens – which appear to be in equal use across treatment centers – to reduce the possibility of heterogeneity in treatment effects in patients enrolled in its control arm. However, this isn't without its challenges and is explored further in Appendix 3.
