# Supplementary material for: Adaptive Design for Phase II/III Platform Trial of Lassa Fever Therapeutics
Source: Emerg Infect Dis. 2025 Feb;31(2):e240251. doi: 10.3201/eid3102.240251 (PMC11845141; doi:10.3201/eid3102.240251)
Supplement: Appendix 3 — Design considerations for a portfolio approach to Lassa fever clinical trials. [file 24-0251-Techapp-s3.pdf]

Article DOI: https://doi.org/10.3201/eid3102.240251

Adaptive Design for Phase II/III Platform Trial of Lassa Fever Therapeutics

EID cannot ensure accessibility for supplementary materials supplied by authors. Readers who have difficulty accessing supplementary content should contact the authors for assistance.

Appendix 3

Design considerations for a portfolio approach to Lassa fever clinical trials

The below four scenarios were considered to evaluate ribavirin before or as a part of a Phase III platform trial of multiple therapeutics in which using placebo in the control arm is considered unacceptable or infeasible.

Scenario 1 – Ribavirin is selected as an active control in a Phase III platform trial

A ribavirin regimen will be selected as the control arm in Phase III comparisons within a platform trial based on (i) ongoing pharmacokinetics studies or (ii) a small clinical trial that separately compares the effectiveness of the two regimens. No comparisons between ribavirin and placebo would take place and therefore no assessment of efficacy can be made. Investigational products in a platform trial would be compared to the best performing ribavirin regimen following one of the abovementioned evaluations. This approach parallels the approach used in a recent Ebola Virus disease trial where an active treatment was used as control (*1*).

In this scenario, a superiority evaluation would be the favored approach as non-inferiority assessment to a treatment without established efficacy data would not be appropriate. Design options for the selection of the ribavirin regimen include a triangular design, with selection based on highest success frequency if both regimens cross the pre-defined success threshold; and a double triangular design for direct comparison between regimen efficacies.

Scenario 2 – The efficacy of ribavirin is established in a three-arm trial (A v. B v. A+B) before a Phase III platform trial can commence

As in Scenario 1, a ribavirin regimen would be selected for the control arm in a platform trial. Before the platform trial commences, however, an assessment of the efficacy of ribavirin

would be performed first using an approach developed for Ebola virus disease (*2*). This would involve a three-arm trial, where patients would be randomized to receive (i) ribavirin, (ii) another investigational product, (iii) both ribavirin and the other investigational product.

In this design, the comparison that informs on the efficacy of ribavirin is between arms (ii) and (iii). This design has strengths: it would allow in a single trial efficacy assessment for both ribavirin and the other investigational product. One potential weakness of this approach is that its validity rests on the assumption of no interaction between treatment effects on the additive scale. The benefit of this general approach is that if future trials show harm of ribavirin, rather than benefit, it would not be necessary to repeat comparisons of investigational products against placebo arm.

Scenario 3 – Standard of care as the control arm in a Phase III platform trial

Another option is to use the standard of care as the control in Phase III comparisons and in comparative Phase II components, which might include ribarivin or not depending on local practices and ribavirin availability. This approach would not directly assess the question of ribavirin efficacy, but it would potentially increase participation in the trial as current standard of care could be continued. If an investigational product, in combination with standard of care, is shown to be superior compared to standard of care alone, a direct comparison between this drug and ribavirin is achievable.

Scenario 4 – No control arm in a Phase III platform trial

In addition to the scenarios described above, all of which involve pairwise comparisons between the experimental arms against a common control arm, another option is the design proposed by Magaret and colleagues (*3*) that assumes there is no control arm and that allow for the identification of the best treatment among those at the beginning of the trial. This design minimises the number of comparisons between arms by, at each interim analysis, ranking arms and performing selected comparisons based on pre-defined rules. One advantage of this design is that it does not require definition of a control arm. However, the publication describing the methodology does not consider the later addition of arms, which is an integral part of the platform trial design in the other three scenarios. Another factor to consider here is the time to first decision as this trial terminates either due to futility or when a single best arm is identified,

which might be different on average from the time to first decision using designs that focus on pairwise comparisons.

References

1. Mulangu S, Dodd LE, Davey RT, Jr., Tshiani Mbaya O, Proschan M, Mukadi D, et al. A randomized, controlled trial of Ebola virus disease therapeutics. N Engl J Med. 2019;381:2293–303. https://doi.org/10.1056/NEJMoa1910993

2. World Health Organization. Note for the Record: consultation on clinical trial design for Ebola virus disease (EVD) [cited 2024 Feb 20]. https://www.who.int/csr/resources/publications/ebola/NFR-study-design-EVD-20-june.pdf

3. Magaret A, Angus DC, Adhikari NKJ, Banura P, Kissoon N, Lawler JV, et al. Design of a multi-arm randomized clinical trial with no control arm. Contemp Clin Trials. 2016;46:12–7. PubMed https://doi.org/10.1016/j.cct.2015.11.003
